# Proteomics-based metabolic modeling and characterization of the cellulolytic bacterium *Thermobifida fusca*

**DOI:** 10.1186/s12918-014-0086-2

**Published:** 2014-08-13

**Authors:** Niti Vanee, J Paul Brooks, Victor Spicer, Dmitriy Shamshurin, Oleg Krokhin, John A Wilkins, Yu Deng, Stephen S Fong

**Affiliations:** 1 Virginia Commonwealth University, Richmond, USA; 2 University of Manitoba, Winnipeg, Canada; 3 Kansas State University, Olathe, USA

**Keywords:** Metabolic Modeling, Flux Balance Analysis, Constraint Based Modeling, Actinomycete, Thermobifida fusca, Proteomics Profiling, Terpenoids Biosynthesis Pathway, DXP Pathway, Mevalonate Pathway, Biofuel

## Abstract

**Background:**

*Thermobifida fusca* is a cellulolytic bacterium with potential to be used as a platform organism for sustainable industrial production of biofuels, pharmaceutical ingredients and other bioprocesses due to its capability of potential to convert plant biomass to value-added chemicals. To best develop *T. fusca* as a bioprocess organism, it is important to understand its native cellular processes. In the current study, we characterize the metabolic network of *T. fusca* through reconstruction of a genome-scale metabolic model and proteomics data. The overall goal of this study was to use multiple metabolic models generated by different methods and comparison to experimental data to gain a high-confidence understanding of the *T. fusca* metabolic network.

**Results:**

We report the generation of three versions of a metabolic model of *Thermobifida fusca* sp. XY developed using three different approaches (automated, semi-automated, and proteomics-derived). The model closest to *in vivo* growth was the proteomics-derived model that consists of 975 reactions involving 1382 metabolites and account for 316 EC numbers (296 genes). The model was optimized for biomass production with the optimal flux of 0.48 doublings per hour when grown on cellobiose with a substrate uptake rate of 0.25 mmole/h. *In vivo* activity of the DXP pathway for terpenoid biosynthesis was also confirmed using real-time PCR.

**Conclusions:**

*i*Tfu296 provides a platform to understand and explore the metabolic capabilities of the actinomycete *T. fusca* for the potential use in bioprocess industries for the production of biofuel and pharmaceutical ingredients. By comparing different model reconstruction methods, the use of high-throughput proteomics data as a starting point proved to be the most accurate to *in vivo* growth.

## Background

With ongoing research in genomics, metagenomics, and bioprospecting, the breadth of novel and interesting biochemistry continues to grow. One of the current challenges associated with the large amount of data and resources available is to conduct detailed analyses to curate information to translate raw data into knowledge that provides functional insight. One computational method that facilitates metabolic analysis and dovetails well with genomic and biochemical information is genome-scale constraint-based modeling. While constraint-based models have benefits of being easily scalable and providing gene-protein-reaction level specificity, there are a number of limitations. One of the most fundamental problems revolves around the fact that metabolic networks are underdetermined and thus, there exist alternative flux states with different pathway usage that produce indistinguishable cellular phenotypes. This is an underlying problem with constraint-based models that impacts multiple facets of these models including the initial reconstruction (association of specific reactions with annotated genes) to producing simulation predictions (presence of alternate optimal solutions). In this study, we consider the metabolically under-characterized actinobacterium, *Thermobifida fusca*, and utilize three different methods to gain a better understanding of its metabolic network and identify preferred methodologies for network characterization.

### Thermobifida fusca

Within the actinomycetes, *Thermobifida fusca* (aerobic, thermophilic, gram-positive) is known for its high temperature and pH stability as well as highly expressed cellulolytic system. The cellulolytic system is comprised of three endocellulases (Cel9B, Cel6A and Cel5A), two exocellulases (Cel6B and Cel48A) and a processive cellulase (Cel9A) [1]. Numerous studies in the past have reported on various facets of the ability of *T. fusca* to degrade lignocellulosic biomass. Due to the high efficiency with which *T. fusca* can process lignocellulosic materials, efforts have been made to clone several individual cellulase genes into *Streptomyces lividians, Streptomyces albus, Bacillus subtilis*[2] and *Escherichia coli*[3],[4]. The cloned enzymes were isolated in good concentrations but failed to show similar level of cellulolytic activity as is found in *T. fusca*. This may be due to the complexity of cellulose degradation systems that are not defined by a few genes but is an intertwined network of various enzymes [1],[5]. Hence, it is suggested that the best way to fully utilize the cellulolytic capabilities of *T. fusca* may be to develop *T. fusca* rather than trying to move its cellulases into other systems by heterologous expression.

**Figure 1:**
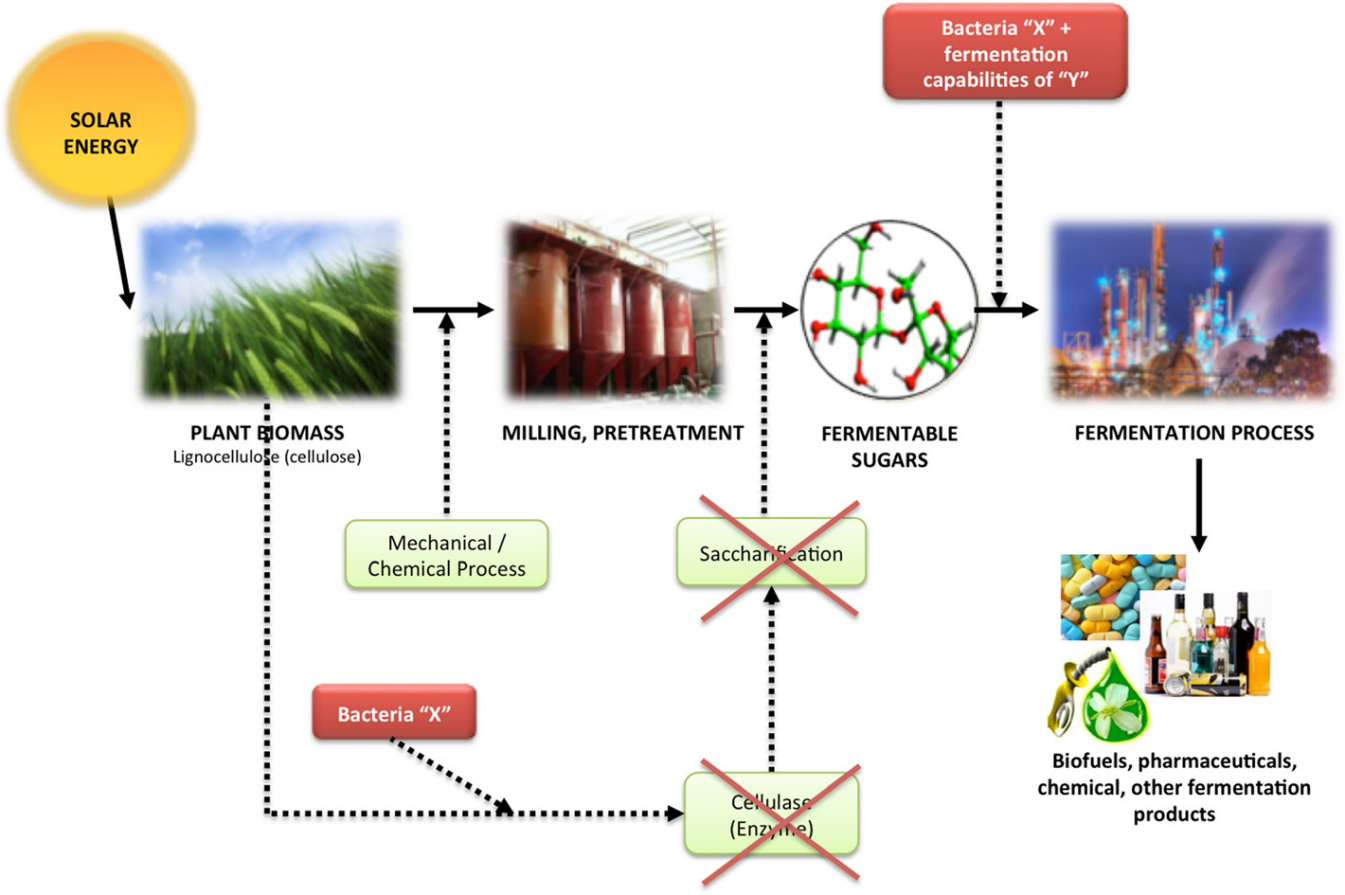
**The prospective use of cellulolytic microbes such as T. fusca may help in reducing the multiple steps towards the fermentation bioprocess there by reducing the need of pretreatment of plant biomass to feed into an industrial process as a source of carbon.** Bacteria-X denotes the cellulolytic capabilities of microbes such as T. fusca and Bacteria-Y denotes the fermentations potentials of industrial microbes

Actinomycetes have historically been involved in the biological production of a variety of antifungals, antibiotics, and chemotherapeutics. Some of these compounds include siderophores [6], polyketides [7],[8] and terpenes [9],[10]. As a cellulolytic actinomycete, *T. fusca* may present an interesting opportunity for consolidated bioprocessing - CBP (Figure 1) of raw lignocellulosic material to value-added biobased products.

The ability to produce chemicals of industrial importance using inexpensive lignocellulosic biomass has been a recent focus for microbial systems. For *T. fusca*, the sequencing of its genome by Department of Energy (DOE) in 2005 sets up a milestone towards understanding this industrially applicable microbe [11]. Besides proving an excellent host microbe for biofuel production [12], it also showed success towards utilization of untreated (without any preprocessing) lignocellulosic material. This is a promising development toward making use of the cellulolytic capabilities of this microbe to reduce the complex multi-step bioprocess to a CBP [13].

Different approaches have been taken in the past in trying to develop a consolidated bioprocess. One approach is to utilize the cellulolytic capabilities to well-established model organisms, such as *E. coli* and *S. cerevisiae*[1]. This approach seeks to allow for direct use of lignocellulosic biomass as a starting point, but leverages the knowledge and tools available for well-characterized organisms. The alternative approach is to characterize and develop poorly-characterized cellulolytic organisms. Thus, high levels of cellulose processivity can be achieved, but the challenge is to develop the knowledge and tools to a sufficient level that metabolism can be designed and altered in a directed fashion.

In studying *T. fusca*, we believe that it can be developed into a facile cellulolytic system for consolidated bioprocessing. In 2011, Deng and Fong established a genetic modification protocol for genetic engineering of *T. fusca* and demonstrated this system through the production and optimization of propanol [12]. The initial developments in the characterization of *T. fusca* are listed in Table 1. Following along the same lines, we continue to further understand the other capabilities of robust cellulolytic system of actinomycete for use in manufacturing industries. Thus, our group aims to present a systems level understanding of metabolic network of *T. fusca*.

**Table 1:** Significant milestones for T. fusca research and characterization

**Year**	**Development**	**Group**	**Citation**
1998	Genus Constructed	Zhang et al.	Int. J Syst Bact. 1998 Apr
2002	Physical Characterization	Kukolya et al.	Int. J Sys Evol Micr, 2002 Jul
2005	Genome Sequenced	DOE	JGI Finished Genome, 2005
2004-2006	Plant biomass degradation study and analysis of enzymatic system	Wilson et al.	Chem Rec. 2004
			Biochem. 2006 Nov
2007	Sequence Annotation	Lykidis et al.	J. Bacteriol. 2007 Mar
2010	First Genetic Modification	Deng & Fong	Appl. Environ. Microbiol 2010 Apr
2011	Producing biofuel from untreated biomass	Deng & Fong	Metab Eng. 2011 Sept

### Metabolic models

With the availability of genomic sequences, it has become possible to use genome annotation and biochemical information to reconstruct cellular metabolic networks [14]. These models can be used for simulating the living state of bacteria, if operated under the defined constraints and boundary conditions. There are various algorithms such as FBA - flux balance analysis [15],[16], MOMA - minimization of metabolic adjustments [17], ROOM - regulatory on-off minimization [18] and MCA - metabolic control analysis that are currently used for the purpose of running computational simulations of these models [19]. In the current study, we used FBA, which is based on linear programming, to simulate and optimize *T. fusca* model developed in this study for biomass production (growth).

### *T. fusca* model and FBA

**Figure 2:**
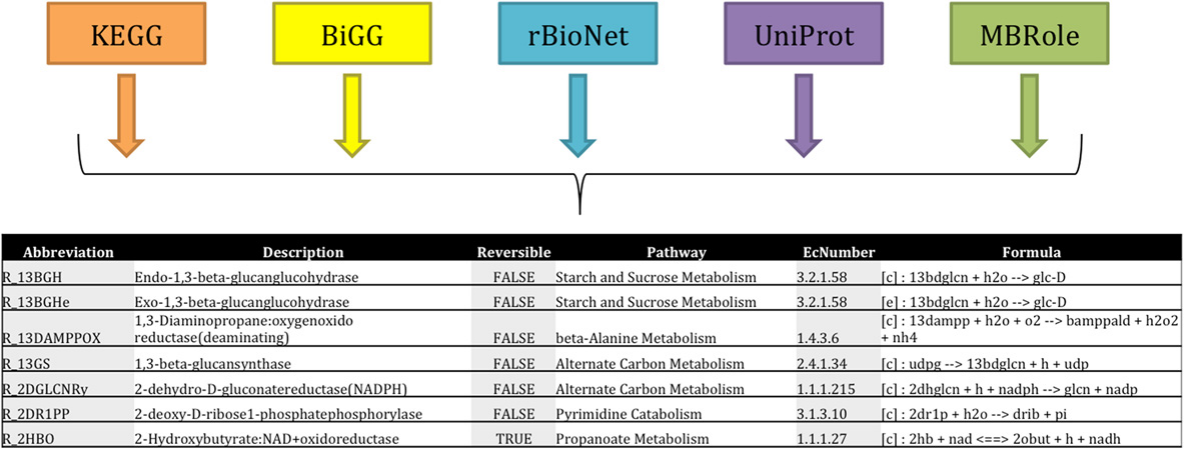
Illustration of available database used for compiling the MetModel reaction database.

Whole genome sequence and annotation of *T. fusca*[11] was used to build a genome-scale metabolic reconstruction to understand the underlying metabolic pathways and network in *T. fusca*[11],[12],[20]. The reaction database used for drafting the model includes but is not limited to sources such as KEGG [21],[22], BiGG [23], rBioNet [24], UniProt [25] and MBRole [26]. Information from disparate databases were tabulated to form an in-house reaction database as illustrated in Figure 2.

The process of compiling biochemical reactions leads to the formulation of a draft model of *T. fusca*, however, at this stage the represented metabolic network inevitably is incomplete and has numerous metabolic gaps. Thus, any initial model that is generated undergoes a gap-filling step. For our models, gap-filling was initially performed using a computational gap-filling algorithm [27]-[30]. After gap-filling, simulations can be run by specifying input constraints such as substrate uptake rate or oxygen uptake rate. Once the model provides a framework to understand the cellular process, it can be used to find target for focused metabolic engineering to yield products of biotechnological value [31]. The most widely-used algorithms for design and simulation of genome-scale constraint-based metabolic models such as OptKnock [32], OptForce [33], EMiLio [34] are based on flux distribution and flow through chemically balanced reactions (FBA). Flux balance analysis (FBA) uses linear programming to optimize the objective function as follows:

(1) $$\begin{array}{l} \mathrm{Maximize}:\;Z\\ \mathrm{Subject}\;\mathrm{to}:\;\mathrm{Sv}\;=\;0,\\ a_{i}\leq v_{i}\leq \;b_{i}\;\mathrm{for}\;\mathrm{all}\;\mathrm{reactions}\;i, \end{array}$$

where, *Z* is the flux through objective function (biomass production and product optimization), S: stoichiometry of the reactions represented as matrix, *v* is reaction flux vector, *a*_*i*_ and *b*_*i*_ are the constraints placed on the flux *v*_*i*_ of the reaction *i*[35].

Even after compiling biochemical information and gap-filling a model, there often are discrepancies between the computational model results and *in vivo* states that are difficult to identify using only computational approaches. An additional level of model curation can be achieved by integrating high-throughput experimental data with the framework of a computational model to put “content in context” [35]. This data integration can be done using multi-scale high throughput experimental data such as transcriptomics, proteomics and metabolomics. This step reconciles *in silico* predictions with experimental results and thereby helps enhance the characterization of the cellular activity.

Genome-scale metabolic models have been created for many prokaryotic microbes and a variety of applications [23]. Several of these models have been able to incorporate experimental data to more closely match cellular processes. Once the model closely resembles a biological system, it can be optimized for defined objective function. This objective function may range from production of biomass to production of a chemical target. Following the *in silico* optimization of yields the computational design may eventually be replicated for applications in industry, therapeutics or health-related predictions.

In this study, three different approaches for generating constraint-based models were used and analyzed for their ability to accurately predict cellular growth when given an input substrate uptake rate. The three model versions were 1) designed using Model SEED [36], 2) an in-house semi-automated reconstruction based on organism specific annotation and reaction information from KEGG [21],[22], and 3) a proteomics-based model from 2D proteomic experiment of *T. fusca* grown on cellobiose media. A comparative analysis between the different models was conducted to develop the most experimentally accurate model of *T. fusca* and to provide comparison of different model generation methods. Detailed analysis of metabolic function of *T. fusca* is also included to provide perspective on potential industrial applications (eg: in biofuel and natural product) for development of consolidated bioprocesses using *T. fusca*. Here we will be discussing the mevalonate and non-mevalonate pathways for terpenoid biosynthesis. These pathways are utilized for the production of isoprenoid precursor (isopentenyl pyrophosphate and dimethylalyl pyrophosphate) compounds that have applications in pharmaceutical, nutraceutical and perfume industries.

## Results and discussion

**Figure 3:**
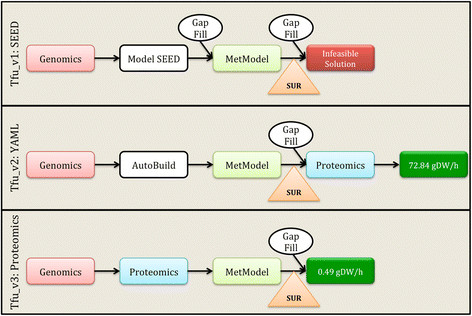
**Flowchart of the model building step and data incorporation for all the three version of the model.** Genomics and Proteomics are the high throughput datasets used for building the models. SUR – Substrate uptake rate for cellobiose as calculated experimentally was applied as a constraint. Red indicated no solution was obtained when running simulation of SEED model under experimental constraints. Values in the green boxes indicate the growth rates for Tvu_v2 and Tfu_v3.

*T. fusca* is known to directly use lignocellulosic biomass as a carbon substrate due to its well-studied cellulolytic system [1],[37]. In laboratory conditions, *T. fusca* can grow on a variety of substrates including glucose, cellobiose, and microcrystalline cellulose. To match experimental work conducted as part of this study on cellobiose, it was necessary to add cellobiose degradation reactions to the draft model based upon the annotation and experimental evidence (EC 3.2.1.21, Tfu_0937). Three versions of a *T. fusca* model were created, as shown in Figure 3, using three different approaches to compare model-building approaches and models were tested against experimental growth on cellobiose. In the process of model reconstruction, activity of terpenoid pathways was uncertain, but was experimentally verified using real-time PCR.

### Metabolic reconstruction: summary and model statistics

Three different draft metabolic models were constructed for *Thermobifida fusca*. The three models varied based upon what was used as the starting point for generating the initial reaction list for the model (Model SEED, KEGG, proteomics data). In all cases, after initial generation of a reaction list, all reactions were associated with KEGG IDs to standardize comparisons between models.

**Tfu_v1:** The taxonomy number of *T. fusca* was used to generate a draft model from Model SEED (Tfu_v1) [36],[38]. The Model SEED output was then converted to KEGG compound identifiers before running gap analysis and FBA. The Model SEED-derived Tfu_v1 model contained 1302 reactions, 1213 metabolites, and 618 EC numbers. Gap analysis added 146 reactions that primarily include a variety of exchange reactions (reactions that denote the direct uptake/secretion of the respective metabolite from or to the extra cellular media). When used with unconstrained carbon input flux (cellobiose uptake of 1000 mmoles/gDW/h) the Tfu_v1 model calculated a growth rate of 24.25 doublings/h. However, when the experimentally determined substrate uptake rate of cellobiose (0.25 mmoles/gDW/h) was applied to this model it failed to arrive at a viable solution (warning of infeasible solution). This infeasibility was crosschecked and verified by using the Model SEED FBA runs. The model failed to perform under any media formulations (glucose and cellobiose) attempted using the Model SEED simulation platform.

**Tfu_v2:** The second version of a *T. fusca* model (Tfu_v2) was created using an in-house semi-automated reconstruction method as defined in our past publications [27],[28]. This model consists of 1002 reactions involving 584 EC numbers and accounting for 1105 metabolites. Eight out of 48 reactions added in the gap analysis were non-exchange reactions. Applying the experimentally determined constraint of substrate uptake rate for cellobiose as 0.25 mmoles/gDW/h, the optimal biomass growth was 72.84 doublings/h. This growth rate was compared to experimental growth rate of 0.43 doublings/h.

**Figure 4:**
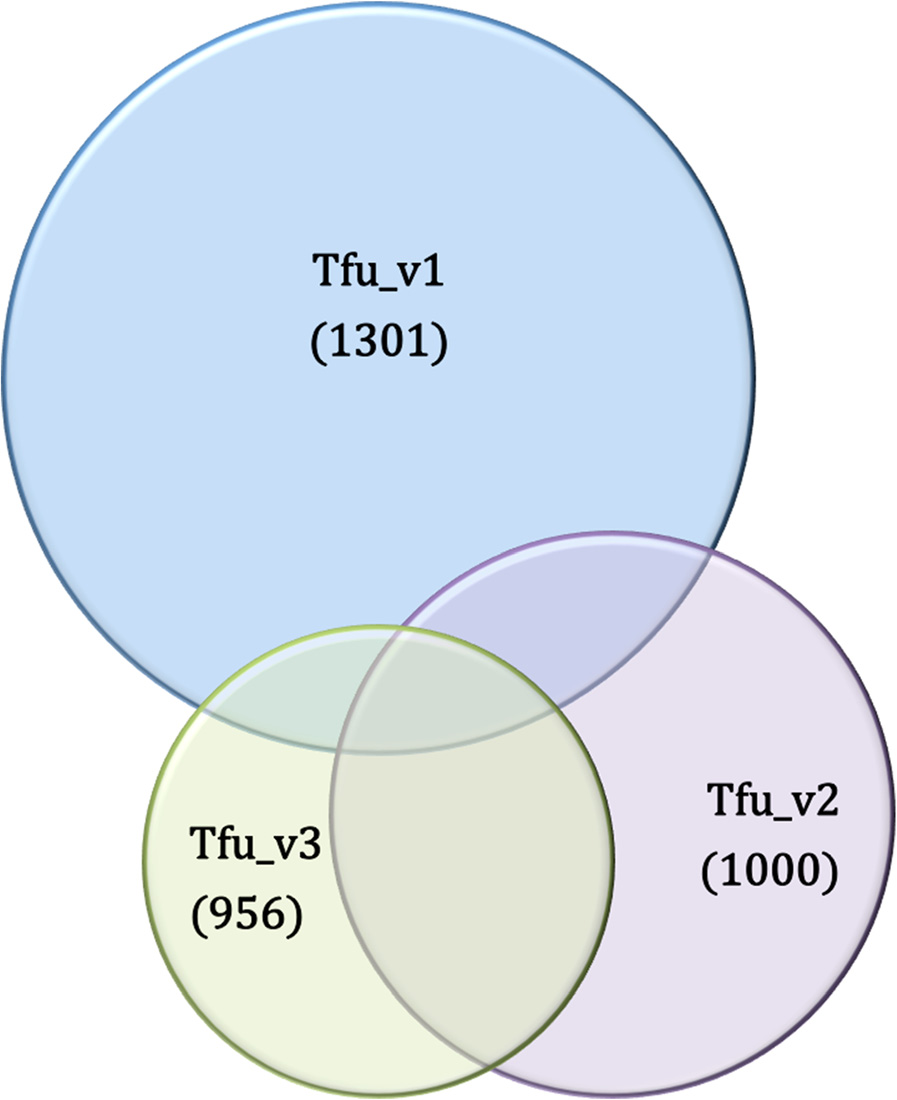
**Summary of three versions of models created in this study: Reaction distribution across the 3 versions is represented in the figure.** All three versions had 170 reactions in common. Following was the common reaction across each compared pair of model versions: Tfu_v1: SEED AND Tfu_v2:yaml: 195, Tfu_v2:yaml AND Tfu_v3: Proteomics: 417, Tfu_v1: SEED AND Tfu_v3: Proteomics: 185.

**Tfu_v3:** The third version of the model generated in this study (Tfu_v3) is based on proteomic experimental data generated by our group in collaboration with the Manitoba Centre for Proteomics & Systems Biology, the University of Manitoba. The detailed protein characterization of *T. fusca* based on this data is beyond the scope of this paper and will be explained in future publications along with the other experimental data. This method of generating a model varied from Tfu_v1 and Tfu_v2 as the initial reaction list was populated from reactions associated with experimentally detected proteins, not informatics-based annotation. The Tfu_v3 model contains 975 reactions, 1382 metabolites, and 316 EC numbers (296 unique genes annotated in *T. fusca*). FBA simulations with an input constraint of a cellobiose uptake rate of 0.25 mmoles/gDW/h gave a predicted growth rate of 0.49 doublings/h (for reference experimentally determined growth rate is 0.43 doublings/h). A comparison of the model contents (by reaction) is summarized in Figure 4 and the detailed model in all three versions is attached as Additional file 1 (Model.xls).

Due to the critical role of central metabolism to heterotrophic organisms, an analysis was conducted using all three model versions to study central metabolism. To study the individual pathways in carbohydrate metabolism and amino acid metabolism, each metabolic pathway was annotated using EC numbers recorded in KEGG. The summary table is shown in Table 2. Genes within a pathway were tabulated to evaluate the completeness of given pathway. At the first glance, all the pathways with a gene presence of 20% or above were counted as present/active.

**Table 2:** Summary of individual pathway contrasts between three versions of model: The reference frames selected here is KEGG

	**KEGG reference**	**In model**					
	**Reactions in pathway**	**Tfu_v1**		**Tfu_v2**		**Tfu_v3**	
**Carbohydrates Metabolism**							
Glycolysis/Gluconeogenesis	45	28	62%	22	49%	15	33%
Citrate Cycle (TCA Cycle)	22	15	68%	15	68%	13	59%
Pentose Phosphate Pathway	40	20	50%	15	38%	13	33%
Pentose and Glucuronate Intrconv.	61	10	16%	8	13%	6	10%
Fructose and Mannose Metabolism	65	14	22%	14	22%	5	8%
Galactose Metabolism	38	12	32%	13	34%	8	21%
Starch and Sucrose Metabolism	74	14	19%	14	19%	12	16%
Amino Sugar and Nucleotide Metabolism	108	25	23%	21	19%	15	14%
Pyruvate Metabolism	64	26	41%	17	27%	15	23%
Glyoxylate and Dicarboxylate Metabolism	66	12	18%	11	17%	7	11%
Propanoate Metabolism	47	17	36%	14	30%	12	26%
Butanoate Metabolism	50	17	34%	12	24%	7	14%
C- 5 Branched Diabasic Acid Metabolism	18	3	17%	3	17%	1	6%
Inositol Phosphate Metabolism	43	4	9%	5	12%	4	9%
**Amino Acid Metabolism**							
Ala, Asp and Glu Metabolism	43	21	49%	19	44%	18	42%
Gly, Ser and Thr Metabolism	63	11	17%	13	21%	9	14%
Cys and Met Metabolism	66	8	12%	3	5%	3	5%
Val, Leu and Ile Degradation	35	18	51%	13	37%	16	46%
Val, Leu and Ile Biosynthesis	14	10	71%	10	71%	7	50%
Lys Biosynthesis	30	13	43%	10	33%	8	27%
Lys Degradation	54	7	13%	8	15%	7	13%
Arg and Pro Metabolism	104	32	31%	26	25%	22	21%
His Metabolism	37	12	32%	13	35%	10	27%
Tyr Metabolism	66	11	17%	8	12%	9	14%
Phe Metabolism	70	11	16%	9	13%	9	13%
Trp Metabolism	69	14	20%	14	20%	9	13%
Phe, Tyr and Trp Biosynthesis	37	26	70%	16	43%	16	43%

Each of these pathways were individually screened to make a call for active or inactive by tracing through the List of EC numbers on the KEGG map. If majority (almost all) of the EC number continuously connecting the pathway were present then the pathways was marked active else inactive.

### Tfu_v3: proteomics model reaction distribution

Based upon the initial model contents and growth rate simulation results, the the Tfu_v3 appears to most closely predict experimental results. Thus, further detailed computational analyses *of T. fusca* are based on the Tfu_v3 model. The Tfu_v3 model is designated as iTfu296 (represents 296 annotated genes).

**Figure 5:**
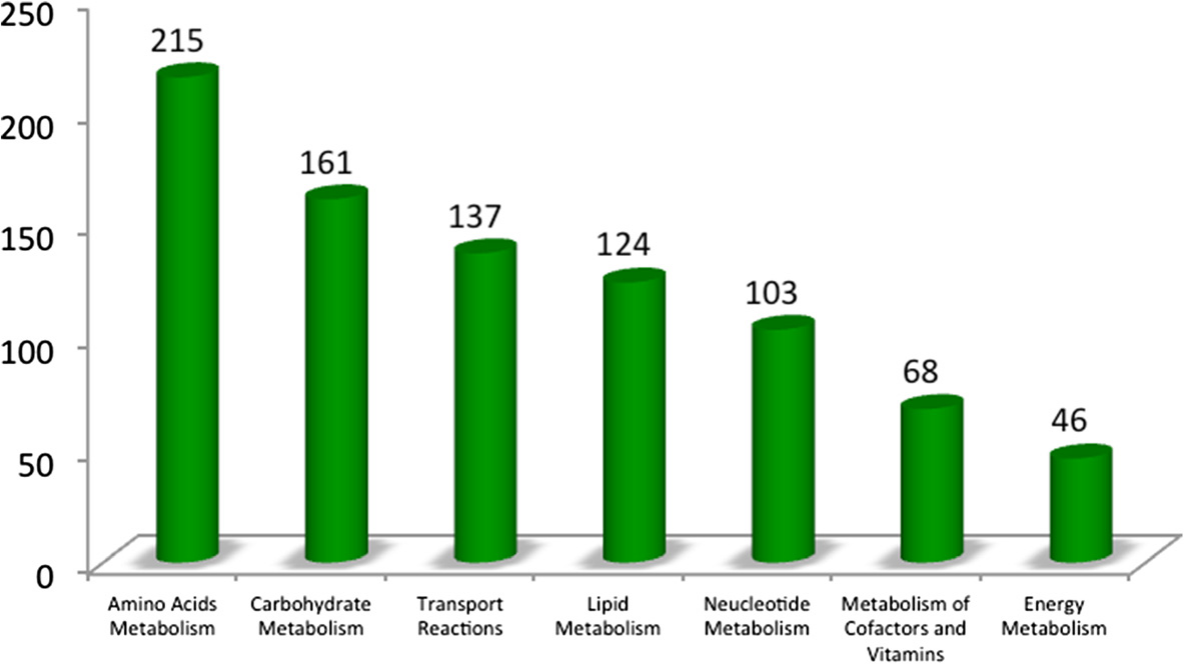
Major reaction distribution in the proteomics based model comprising total of 1002 reactions.

The proteomics-based model of *T. fusca* accounts for 216 amino acid reactions and 182 carbohydrate-related reactions as illustrated in Figure 5. When running an FBA simulation to maximize growth with an input cellobiose uptake of 0.25 mmoles/gDW/h, 110 reactions were highly active as indicated by high calculated fluxes (flux values of >100 or < −100). (The range of flux through reactions was from 0 to 1000; with a threshold of 100 arbitrarilty selected for the purpose of analysis). Among these 110 reactions, the majority of the reactions were used in carbohydrate (37) and amino acid (34) metabolism (i.e. 60 unique reactions after eliminating overlaps between carbohydrates (37) and amino acid (34)).

**Figure 6:**
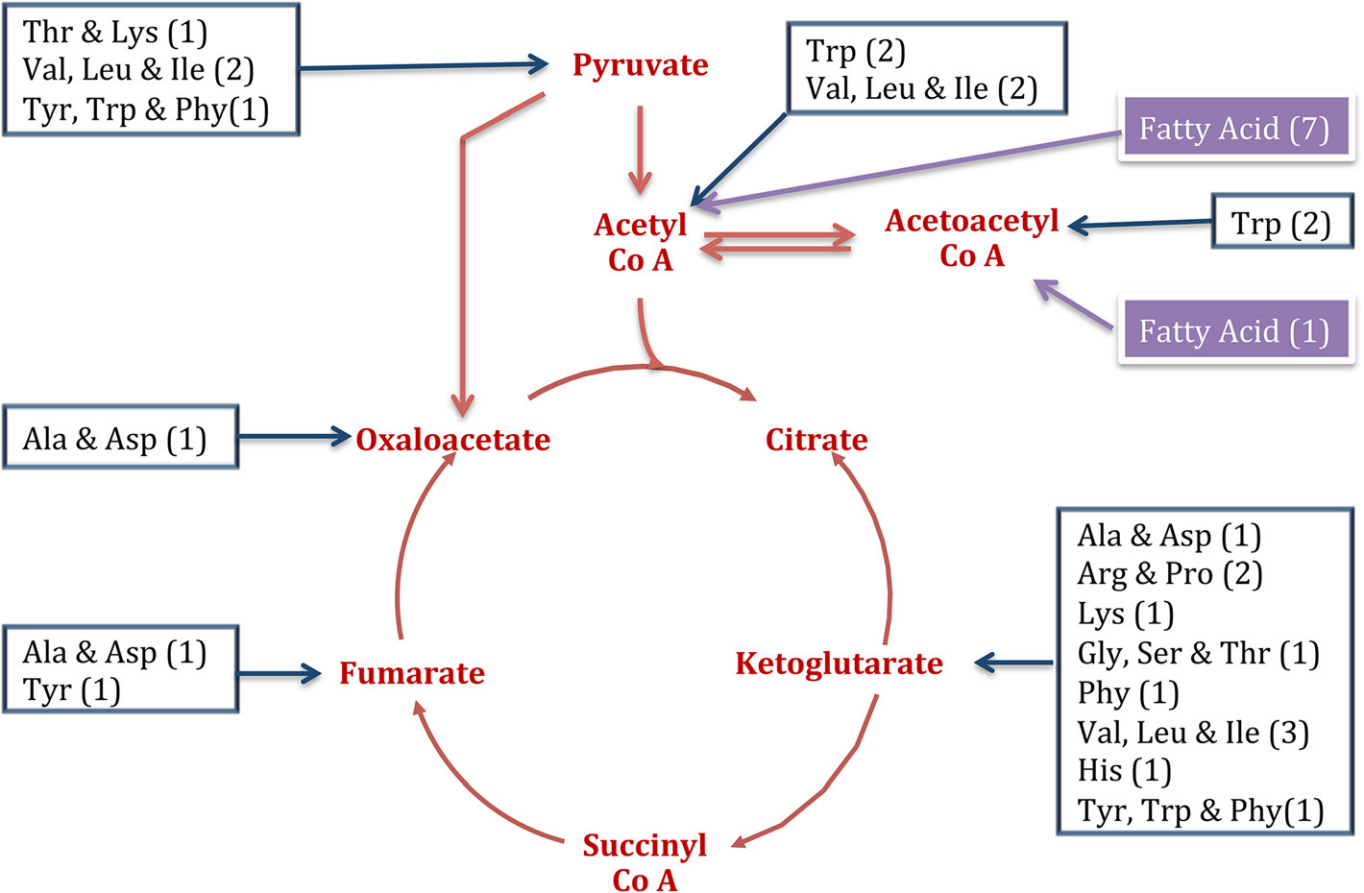
**Summary of carbohydrate and amino acids connection nodes and number of active flux reaction within amino acid pathways.** Standard three-letter amino acids code is used to denote the pathway names. The carbohydrate nodes of the central metabolism are highlighted as red and the branching out amino acid pathways are boxed in blue. The purple boxes denote the fatty acids connections from Acetyl Co A and Acetoacetyl Co A. The numbers of reactions for each pathway are represented in the parenthesis.

In carbohydrate metabolism, the tricarboxylic acid cycle (TCA cycle) was complete and active. In the current study, we will be focusing on the seven compounds of TCA cycle that play crucial roles in carbon exchange between various pathways in the central metabolism. These intermediates include: pyruvate (KEGG compound C00022), acetoacetyl CoA (C00332), acetylyl CoA (C00024), alpha-ketoglutarate (C00026), succinyl CoA (C00091), fumarate (C00122) and oxaloacetate (C00036). Studying the reaction use and fluxes involving these compounds will help us understand the probable activity in amino acid biosynthesis and degradation pathways. To summarize these nodes in the current proteomics-based version of our model, 60 unique reactions (associated with carbohydrates and amino acid metabolism) were found with some significant flux (i.e. flux was > 0.01 units). Among these, 23 unique reactions correspond to amino acid metabolism. This indicates the activity around the most of the amino acid – carbohydrate connection nodes as shown in Figure 6.

It was observed that in the carbohydrate-amino acid network connection the majority of reactions (11 out of 23) branch in and out of alpha – ketoglutarate. The acetyl CoA node was more centralized by connecting the amino acids and fatty acid pathways with 8 significantly high flux reactions. The pathway specific details are also illustrated in the Table 2. It was observed that when contrasted with the reactions specific to the biosynthesis or degradation of amino acids, the overall analysis suggests the presence of all but one amino acid subsystem, cysteine and methionine metabolism.

### Comparison of “model refinement with data” Vs “reconstruction from data”

In the past, several attempts have been made to use computational metabolic models as a scaffold for experimental data integration [39]-[42]. Experimental data integration not only serves as a means of testing model predictions, but can also be used to help refine the model solution space to provide simulations that more closely match *in vivo* flux states. In this study, we used two methods to combine metabolic models and experimental data to understand and characterize the metabolic network of *T. fusca*. For the first approach, an MILP algorithm (analogous to Shlomi et al. [42]) was used to integrate the proteomics dataset to the model Tfu_v2 (autobuild model). This algorithm aims at optimizing the agreement between the experimental data and the *in silico* model [42]. In this context, the experimental information is used to assign a present or absent call to re-channelize the flux distribution of the network as explained by Gowen et al. [43].

Parallel to this, a second approach originating directly from an experimental proteomic data set was used to generate an independent model, Tfu_v3. Currently existing methods for constraint-based model reconstruction primarily depend on the bioinformatic information such as genomics data, biochemical data and models of related microorganisms at the initial phase of model building. The approach taken for construction of Tfu_v3 relies on the *in vivo* experimental evidence of the proteins as the starting point of model building. While these two methods of model construction used the same bioinformatics and experimental data, subtle differences in the order of process steps and algorithms, as shown in Figure 5, used to construct the two models resulted in vastly different functional consequences.

Besides this, another very interesting and significant difference between Tfu_v2 and Tfu_v3 was observed in the function of the TCA cycle. The reaction using pyruvate to make oxaloacetate was not found in the autobuilt Tfu_v2 version whereas the proteomics version clearly shows its presence (EC 6.4.1.1, Gene ID: Tfu_2557, Tfu_1530, Tfu_0947, Tfu_1228). In addition, most of the amino acid pathways were fully or partially incomplete in Tfu_v2. Thus, the Tfu_v2 model had artificially high fluxes through transport reactions to uptake external nutrients to satisfy simulation requirements for optimal biomass production. The Tfu_v3 model showed most of the pathways significantly complete except cysteine and methionine metabolism. Besides cysteine and methionine metabolism, phenylalanine metabolism was sparsely populated, but active flux through the reaction involved in the interconversion of phenylalanine to phenylpyruvate suggested making a present call for the entire phenylalanine pathway. These functional differences indicate some of the potential danger associated with over-reliance on genome annotation (that may contain numerous errors especially in under-characterized organisms).

### Applicability of the model: biofuels and pharmaceutical precursors

The subsystem-based analysis of central metabolism and experimental foundation suggests the closer association of Tfu_v3 to the *in vivo* biochemical system of *T. fusca*. While the Tfu_v3 model is the most accurate of the three models constructed in this study, there are numerous areas of metabolism that are not well-characterized. For comparison, even the most update model of *E. coli* has an account of only for 30% of the gene products in the model [44]. Likewise, this model being the first ever *T. fusca* metabolic network also opens huge scope of pathway-focused review and improvement. Once completely functional these models provide a ground for hypothesizing a target for the further study.

*T. fusca* is a potentially interesting organism for biochemical production of sustainable fuels or industrial chemicals. In these areas, two pathways of particular interest are butanol and secondary metabolite biosynthesis (e.g. terpenoids). The Tfu_v2 (ModelSEED autobuilt) model incorporates most of the reactions present in the butanoate metabolism however, no active flux was observed through most of them. Tfu_v3 based on experimental dataset confirms approximately 50% of these reactions but also predicts no active flux through these pathways. Thus, while production of butanol through butanoate metabolism appears possible in terms of biochemical capabilities, it remains to be explored and demonstrated experimentally. For comparison purposes, in 2011 a mutant strain *T. fusca* B6 was designed and constructed with heterologous expression of a bifunctional alcohol dehydrogenase (adhE2) for production of 1-propanol [12]. Engineered production of 1-propanol in *T. fusca* provides first-step experimental evidence that *T. fusca* may be usable for the production of fuels directly from lignocellulosic raw materials.

One consideration for FBA simulations of both butanol/butanoate and terpenoids is that these are secondary pathways and utilization of these pathways are not explicitly included in the biomass objective for growth maximization simulations. Thus, it is not necessarily surprising that growth simulations with no genetic designs incorporated may not show flux through secondary pathways. However, due to the potential for secondary metabolism in actinomycetes, we considered studying the terpenoid backbone (TBB) pathway in more detail.

**Figure 7:**
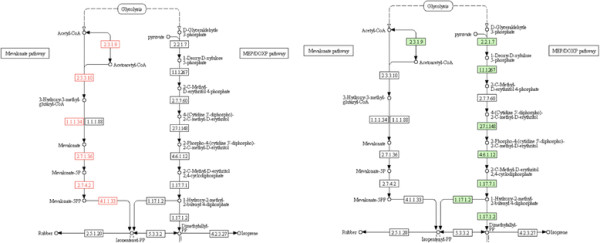
TBB as from flux analysis through Mevalonate Pathway (left) and expression analysis of Non mevalonate pathway (right).

With the aim of characterization, 16 reactions for the TBB pathway were added to the model to computationally test the feasibility of flux through the TBB pathway when optimizing for the biomass production. All the reactions of the mevalonate pathway except R001123 had an active flux of approximately 0.53 mmoles/gDW/h. The reaction R01123 was responsible for isomerization reaction between isopentenyl diphosphate (IPP or C00129) and dimethylallyl pyrophosphate (DMAPP or C00235). The positive flux through the reaction (R01121) lead to the formation of IPP, as shown in Figure 7, and provided computational support that *T. fusca* may possess the capability to divert the flux through the mevalonate pathway reactions. This data was used as a clue to conduct follow-up experiments to verify the active *in vivo* expression of these pathways.

### Experimental validation: expression analysis TBB pathway genes

**Figure 8:**
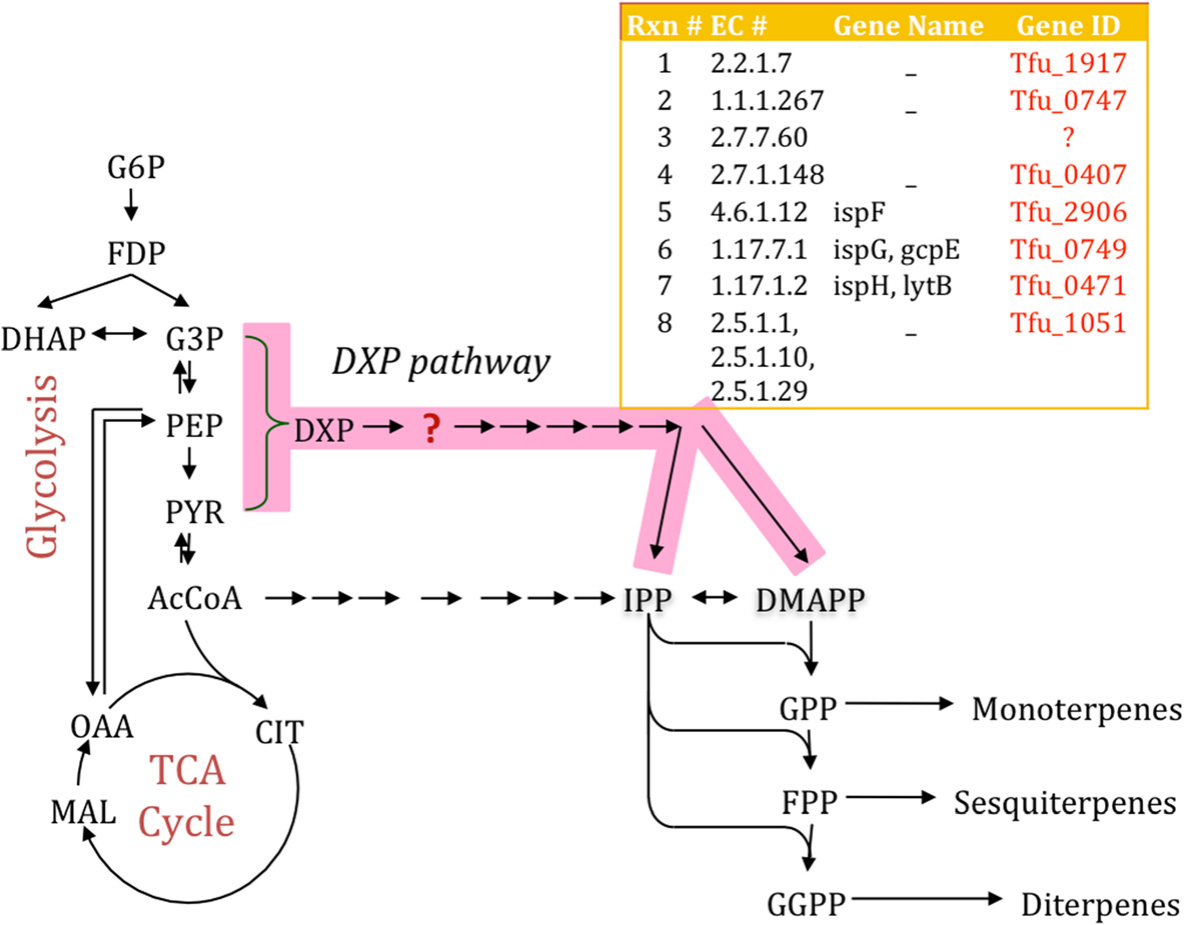
TBB through the non-mevalonate pathway and gene annotation in T. fusca.

Different bacteria have been found to produce terpenoids by either the mevalonate pathway or the non-mevalonate pathway (DXP pathway). While computational simulations demonstrated the feasibility of an active mevalonate pathway, no clear evidence existed for either the mevalonate or the DXP pathways. Given the shorter length of the DXP pathway, initial experimental testing focused on the seven genes of the DXP pathway (Figure 8).

**Figure 9:**
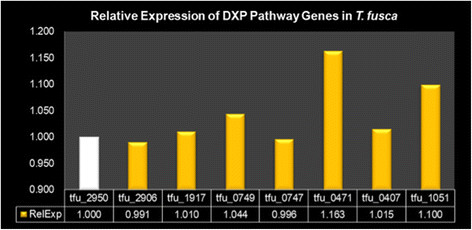
**Relative expression of terpenoid biosynthetic genes associated with the DXP pathway in T. fusca.** Tfu_2950 is a housekeeping gene with constitutive expression in T. fusca.

Real-time PCR was used to measure mRNA transcript levels for the genes in the DXP pathway. The details of *T. fusca* genes studied and tested are listed in Table 3 along with their relative expression in Figure 9. All but one of the required genes were found to be expressed in the strain. The missing enzyme was 2-C-methyl-D-erythritol 4-phosphate cytidylyltransferase (EC: 2.7.7.60). This suggests the presence of terpenoid backbone biosynthesis via the DXP pathway in *T. fusca*.

**Table 3:** T. fusca genes associated with the DXP pathway for terpenoid biosynthesis

**Locus**	**NCBI_GeneID**	**Strand**	**Start Pos**	**End Pos**	**NT seq**
Tfu_1917	3580825	Complementary	2242018	2243931	1914
Tfu_0747	3578952		880259	881473	1215
Tfu_0407	3580006		458401	459324	924
Tfu_2906	3581392	Complementary	3421460	3421924	465
Tfu_0749	3578954		883104	884261	1158
Tfu_0471	3579613		532475	533476	1002
Tfu_3076	3580076		3598495	3599487	993

## Conclusions

Three different methodologies were applied to create metabolic reconstructions for *T. fusca* . The proteomics-based model (Tfu_v3) named *i*Tfu296 was found to mimic the biological growth conditions most closely. It was observed that when cellobiose uptake rate was constraint as 0.25 mmoles/gDW/h gave the growth rate of 0.49 doublings/h. This was comparable to the experimental growth rate of 0.43 doublings/h. This model was built using a novel scheme for model reconstruction based on high throughput proteomic data at the initial model building phase.

Genomic datasets are the most standard high throughput data currently available, but it is always a concern to what extent the genomics information is really transcribed and translated into the functional role inside the cell. Out of 3195 genes annotated in the *T. fusca* genome published in 2005, only 3117 translated into protein-coding genes and only 1757 were associated with predicted functions [11],[45]. This discrepancy between genomic and proteomic information may be due to environmental or evolutionary selection processes. Accurate proteomic data provides more information about the functional activities of the cell, as confirmed by the fact that our proteomics-based model agreed with experimental observations better than models built based on genomic information alone.

With the advent of standard genomics information, genome-scale metabolic models have become a widely used approach to gain a systems level understanding of metabolic processes and function [31]. Every organism-specific model that is built needs to pass through multiple levels of curation and validation based on genome annotation, experimental evidence and (or) biochemical literature study. Knowing that omics-data can be used to help identify *in vivo* function, we applied an approach model building based on high throughput proteomic data. These models contain the scope of integration of genomics, transcriptomics, proteomics, metabolomics and phenomics data. For the current study, proteomics data has been used to establish a significantly reliable starting point for the metabolic model reconstruction. This version of model Tfu_v3 is based on functional building blocks that are more closely associated with the phenotypic characteristics when compared to genomics data in the hierarchy.

In a larger context, the modeling approach aims at establishing links between the molecular and cellular functions. However, it is still hard to find complete agreement between the “biology - biochemistry” and “network models - omics data”. It is reported that the most updated *E. coli*[44] model only associates to 30% of gene products in the model and 1/3 of the gene products are not functionally annotated [46]. Even with this limitation, model-based systems analysis can be useful for developing hypothesis and target for the focused study. In this case, we hypothesize the presence of an active terpenoid backbone pathway, which will be the focus of our follow up studies using experimental and analytical methods.

Nevertheless, this promising approach suffers with a major limitation to date – a lack of a standardized reaction database to build and analyze metabolic network. Due to inconsistency in the labeling of the metabolites (eg: citrate is almost chemically equivalent to citric acid; 2-hydroxy-1,2,3-propanetricarboxylic acid; 2-hydroxytricarballylic acid and have the same compound identifier on KEGG) it is difficult to assemble a non-redundant reaction database with standard nomenclature. Current systems biology experts are in the quest of cleaning and populating available database with minimal redundancy in the hope of exhaustive coverage of cellular biochemical reactions. Some of the examples are MetRxn and MetaCyc.

Computational analysis demonstrated the theoretical feasibility of producing terpenoids in *T. fusca*, however, no existing experimental evidence had previously supported or demonstrated this capability. Analysis of mRNA transcripts showed *in vivo* activity of the DXP pathway in *T. fusca* providing evidence that *T. fusca* may be capable of direct cellulose-to-terpenoid biosynthesis. Isoprene is the monomeric unit for the huge family of terpenoids, thus hold importance in pharmaceutical industry, perfumes, incense, flavoring, spices, and varnishes.

*T. fusca* was found to be a biofuel producing strain after the genetic modification protocol for this strain was established by Deng & Fong. With this systems level characterization of secondary metabolites, it can be suggested as a highly useful, robust and inexpensive strain for industrial application. However, this opens an arena for the scale up and optimization study to successfully launch this strain in industrially significant microbes.

## Methods

### Culture conditions

*Thermobifida fusca* ATCC BAA-629 was grown in Hagerdahl medium containing 1.0% cellobiose. Experiments were conducted in Erlenmeyer flasks where 50 mL pre-cultures of *T. fusca* YX were grown at 55°C and 250 rpm for 24 hours in a 500 mL Erlenmeyer flask. Growth cultures for testing were inoculated using 5% of the pre-culture and grown at 55°C and 250 rpm for 42–48 hours.

### Metabolic network reconstruction

Overall model construction steps illustrated in Figure 3 are as follows:

**Tfu_v1: SEED.** The autobuilt draft model was made in Model SEED [36],[38] and used as the draft network. The *.xml file downloaded was converted into in-house MetModel format to run the FBA simulations with MetModel software.

**Tfu_v2: yaml.** The *.yaml autobuilt drafts were created by our group in 2008 using the then available organism specific annotations and reaction database from KEGG. [27],[29],[43].

**Tfu_v3: Proteomics.** The draft model was constructed using the output of a 2-dimensional LC-MS analysis performed at the Manitoba Centre for Proteomics & Systems Biology (University of Manitoba, Winnipeg, Canada). Cellobiose grown T. fusca sample was subjected to FASP lysis/digestion procedure (J.R. Wisnewski et al. Nature Methods 2009. 6(5). 359–362) followed by 2D-HPLC-MS/MS acquisition using TripleTOF 5600 mass spectrometer (ABSciex, Mississauga, ON) [47]. Thirteen pairwise-concatenated fractions in the first dimension were analyzed over a 1-hour HPLC-MS/MS session, each. This collection yielded 276,129 MS/MS spectra that were interrogated using an in-house GPU-based search engine [48] and yielded identification of 126,471 peptides (16,598 non-redundant) spanning 2101 proteins. This represents approximately 68% total proteomic coverage. Protein identification expectation values were computed using a Bayes’ theorem application of its member peptide expectation values, following the design by Beavis and Fenyo for X!tandem [49]. Over 1700 proteins have expectation values of log(e) < −10 (a one in ten-billion probability of random miss-assignment).

**Figure 10:**
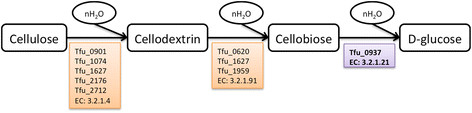
Cellulose degradation reaction in presence of water: The reaction to convert cellobiose as per the experimental dataset Tfu_0937, EC 3.2.1.21 was expressed and thus the associated reaction was added to the model.

Open source *T. fusca* (Taxon identifier: 269800) database KEGG [21],[22], IMG [45] and UniProt [25] was used to map the gene identifiers to the EC numbers which was then looked up as the search identifier in the reaction database compiled in-house. Further, manual curation was done using the published biochemical literature specifically for cellobiose utilization as explained in Figure 10.

### Linear programming for flux balance analysis

In-house python scripts were used to run the FBA simulations using the linear programming algorithm as shown in the introduction.

### Objective function: biomass equation

This draft model aims at the growth optimization and the best estimates used in metabolic modeling scenario are biomass equation, which was designed by slightly manipulating the biomass equations from closely related species and available published information about *T. fusca* growth conditions [50]. The box below shows the biomass equation used for the simulation of all the version of the model. The details of the compounds identifiers are listed in the Additional file 2.

(2) $$\begin{array}{l} \mathrm{BIOMASS}\;\mathrm{EQUATION}\\ 34.7964805\;C00001\;+\;40.1701382\;C00002\;+\;0.00780937\;C00003\;+\\ 0.00780937\;C00006\;+\;0.00780937\;C00010\;+\;0.00780937\;C00016\;+\\ 0.00780937\;C00018\;+\;0.00780937\;C00019\;+\;0.25601316\;C00025\;+\\ 0.00780937\;C00034\;+\;0.59580299\;C00037\;+\;0.00780937\;C00038\;+\\ 0.50005823\;C00041\;+\;0.20910125\;C00044\;+\;0.33354560\;C00047\;+\\ 0.23467873\;C00049\;+\;0.00780931\;C00059\;+\;0.28827498\;C00062\;+\\ 0.12987656\;C00063\;+\;0.25601316\;C00064\;+\;0.20970184\;C00716\;+\\ 0.00780937\;C00070\;+\;0.14934101\;C00073\;+\;0.14026667\;C00075\;+\\ 0.00780937\;C00076\;+\;0.05515731\;C00078\;+\;0.18056213\;C00079\;+\\ 0.13425080\;C00082\;+\;0.08898018\;C00097\;+\;0.00780937\;C00698\;+\\ 0.43865670\;C00123\;+\;0.01174687\;C00131\;+\;0.09262265\;C00135\;+\\ 0.21542571\;C00148\;+\;0.23467873\;C00152\;+\;0.00780937\;C00175\;+\\ 0.41159840\;C00183\;+\;0.24664683\;C00188\;+\;0.00780937\;C00238\;+\\ 0.00780937\;C00255\;+\;0.01174687\;C00286\;+\;0.00780937\;C00305\;+\\ 0.28255111\;C00407\;+\;0.01174687\;C00458\;+\;0.01174687\;C00459\;+\\ 0.09247649\;C04574\;+\;0.00780937\;C14818\;+\;0.00780937\;C14819\;+\\ 0.00780937\;C00229\;-->\\ 40.0000000\;C00008\;+\;39.9921906\;C00009\;+\;0.60239528\;C00013\;+\\ 40.0000000\;C00080\;+\;0.00780937\;C03688 \end{array}$$

### Gap analysis and model comparison

The draft model consists of the list of reactions however there are patches in the network that obstruct continuous flow of flux through the pathway. These links are filled in by using the reaction databank and suggesting the list of reactions required to complete the network. FBA-GAP is used to suggest the connection nodes/reactions that are missing [30]. The use and application of this framework have been described in past by Roberts et al., Gowen et al. and Vanee et al. [27],[29],[43]. FBA-GAP takes a draft model and biomass reaction and uses distances in the reaction network and mathematical optimization to produce a list of metabolites that are necessary for biomass production but cannot be produced or consumed by the cell. Reactions producing and consuming this list of metabolites are obtained from a reference database. These potentially gap-filling reactions were manually checked for relevant evidence such as associated proteins/enzymes characterized or genome annotations,. On detecting the specific evidence these reaction additions to the model wer accepted. The process is repeated until a positive biomass flux value is obtained. In this way, only high-confidence reactions are used to complete the reconstruction.

### Data integrations and model validation for Tfu_v2

The mixed integer linear programming algorithm (MILP) published in 2008 by Shlomi et al. [42] was used for integration of proteomics data to the Tfu_v2 version of model. This algorithm was re-written in python by Gowen et al. [43] to include in our MetModel package.

### Characterization of TBB pathway

Metabolic model *i*Tfu296 or (Tfu_v3) was used to enlist the already existing secondary metabolites production pathway based reactions (characterized by EC numbers) and metabolites (characterized by KEGG compound IDs). In addition, besides studying the complete secondary metabolite network a focused study on terpenoids backbone biosynthesis (TBB) pathway using mevalonate and non-mevalonate pathway was performed using KEGG (map00900). The reactions database of these pathways was created using the information from KEGG as shown in the Table 4 below.

**Table 4:** Reactions associated with Terpenoids backbone biosynthesis pathway that were added to the model Tfu_v3 for the simulation of Secondary metabolites pathway central hub

**Rxn ID**	**Enzyme**	**EC Number**	**Equation**
#Mevalonate pathway			
R00238	acetyl-CoA C-acetyltransferase	2.3.1.9	[c]: C00024 -- > C00332
R01978	hydroxymethylglutaryl-CoA synthase	2.3.3.10	[c]: C00332 + C00024 -- > C00356
R02082	3-hydroxy-3-methylglutaryl-CoA reductase	1.1.1.34	[c]: C00356 -- > C00418
R02245	mevalonate kinase	2.7.1.36	[c]: C00418 -- > C01107
R03245	phosphomevalonate kinase	2.7.4.2	[c]: C01107 -- > C01143
R01121	diphosphomevalonate decarboxylase	4.1.1.33	[c]: C01143 -- > C00129
R01123	isopentenyl-diphosphate delta-isomerase	5.3.3.2	[c]: C00129 -- > C00235
#Non mevalonate pathway			
R05636	DOXP synthase (Dxs)	2.2.1.7	[c]: C00118 + C00022 -- > C11437
R05688	DOXP reductase (Dxr)	1.1.1.267	[c]: C11437 -- > C11434
R05633	MEP synthase (IspD)	2.7.7.60	[c]: C11434 -- > C11435
R05634	CDP-ME kinase (IspE)	2.7.1.148	[c]: C11435 -- > C11436
R05637	CDP-MEP synthase (IspF)	4.6.1.12	[c]: C11436 -- > C11453
R08689	HMB-PP synthase (IspG)	1.17.4.3	[c]: C11453 -- > C11811
R05884	HMB-PP reductase (IspH)	1.17.7.1	[c]: C11811 -- > C00129
R08209	HMB-PP reductase (IspH)	1.17.1.2	[c]: C11811 -- > C00129
R01123	IPP delta-isomerase	5.3.3.2	[c]: C00129 -- > C00235

### Expression analysis of TBB pathway genes

*T. fusca* strain YX grown on Cellobiose media to till the early log phase with dry cell weight of 2.005 mg/mL was used for isolation of RNA using the Qiagen RNA Protect and Qiagen RNAeasy kit. The total RNA was sent to Nucleic Acid Research Facility (Virginia Commonwealth University) for RT-PCR. Tfu_2950 was selected as housekeeping gene to measure the relative expression levels.

## Competing interests

The authors declare that they have no competing interests.

## Authors’ contributions

NV constructed, analyzed and compared the three versions of model. NV and JPB ran the model simulation using MetModel the scripts. NV, VS, DS, OK and JAW worked on proteomics experiments to generate, pre-process and analyze proteomics data. NV integrated the experimental dataset with the model and completed model validation with the programming support from JPB. YD worked on growth curve experimental analysis, biomass equation and first draft of the version one of model. SSF conceived of the study. All authors read and approved the final manuscript.

## Additional files

## Supplementary Material

Additional file 1: **Three_Versions_of_Model.xlxs.** xlxs file with three spreadsheets illustrative the reaction flux in three version of the model. Click here for file

Additional file 2: **Biomass Equation Compound Identifiers.xlxs.** xlxs file with the list of reactants and products on the biomass equation and their description from Kegg database. Click here for file
